# Associations between dietary isoflavones and subtypes intakes and the risk of new-onset of type 2 diabetes mellitus in Chinese adults: a prospective cohort study from the Chinese health and nutrition survey

**DOI:** 10.3389/fnut.2026.1856176

**Published:** 2026-06-16

**Authors:** Yanling Yao, Yichen Geng, Qian Ma, Xiaoling Wang, Huicui Meng

**Affiliations:** 1 Department of Clinical Nutrition, The Eighth Affiliated Hospital, Sun Yat-sen University, Shenzhen, China; 2 School of Public Health (Shenzhen), Shenzhen Campus of Sun Yat-sen University, Sun Yat-Sen University, Shenzhen, China

**Keywords:** Chinese adults, daidzein, genistein, glycitein, isoflavones, type 2 diabetes mellitus

## Abstract

**Background and aims:**

The longitudinal associations between dietary isoflavones and subtype intakes with the incidence of type 2 diabetes mellitus (T2DM) remains poorly understood. This prospective cohort study aimed to investigate the associations between dietary isoflavones and subtypes, including genistein, daidzein, and glycitein, with the risk of new-onset T2DM in Chinese adults.

**Methods:**

A total of 14,652 adults from the Chinese Health and Nutrition Survey (1997–2015) were enrolled. Intakes of isoflavones and subtypes were assessed through three consecutive 24-h dietary recalls and food weighing method. Statistical analysis was performed using a Cox proportional hazards regression model.

**Results:**

A total of 1,051 new T2DM cases were diagnosed during a mean of 10 years of follow-up. In the fully adjusted models, dietary intakes of isoflavones (*p*-trend = 0.0116), daidzein (*p*-trend = 0.0056), glycitein (*p*-trend = 0.0133) and genistein (*p*-trend = 0.0032) were inversely associated with T2DM risk, respectively. This inverse association remained robust in sensitivity analyses. Region modified the associations between dietary glycitein intakes and risk of T2DM. RCS analysis demonstrated low T2DM risk with the intake range of 10.65–24.58 mg isoflavones, 3.80–8.29 mg daidzein, 0.70–2.02 mg glycitein and 4.97–11.31 mg genistein, respectively (*p*-nonlinear<0.0001).

**Conclusion:**

The study provided new evidence for the reference intake of dietary isoflavones and subtypes targeted for the T2DM prevention in Chinese population.

## 1 Introduction

Diabetes mellitus is a cardiometabolic disease with a global prevalence of 11.11% as of 2024, in which over 90% is Type 2 diabetes mellitus (T2DM) accounts, representing a major public health challenge (1). In China, there are almost 233 million patients with T2DM, and the prevalence and incidence continue to rise annually (2, 3). Lifestyle modifications, particularly dietary interventions, are fundamental to the prevention and management of T2DM (4, 5).

Several studies have identified inverse associations between dietary legume intakes with the risk of T2DM (6–8), and this relationship may be attributed to the beneficial effects of nutrients or bioactive components enriched in legumes on glucose metabolism. Isoflavones are phytoestrogens predominantly found in legumes, and their main subtypes include genistein, daidzein, and glycitein. Through their antioxidant, anti-inflammatory, and hormone-regulating properties, isoflavones demonstrate significant potential for chronic disease prevention (9, 10). However, studies assessing the associations between dietary isoflavones intakes and T2DM risk are limited, and available studies are mainly conducted in postmenopausal women in cross-sectional or case–control studies (6, 8). There is a lack of research investigating this topic in prospective cohort studies, and very few studies involve both female and male subjects. In terms of the relationship between isoflavones intakes and T2DM, a randomized controlled trial (RCT) in Qatar has demonstrated favorable HbA1c and insulin profiles following interventions with soy protein and isoflavones in patients with diet or metformin controlled T2DM (11). However, another RCT in Hong Kong, China has reported no significant effect of a diet enrich in isoflavones on blood glucose or insulin concentrations in postmenopausal women with prediabetes or untreated early diabetes (12). These equivocal findings underscore the need for further research on the associations between long-term dietary isoflavones intakes and risk of new-onset T2DM in both men and women. In addition, the relationship between subtypes of isoflavones and risk of T2DM remain unknown.

The aim of this study was to investigate the associations between dietary intakes of isoflavones and subtypes, including genistein, daidzein, and glycitein, with risk of new-onset T2DM in Chinese adults. The hypothesis of the study was that higher dietary intakes of isoflavones and subtypes were inversely associated with risk of new-onset T2DM.

## 2 Materials and methods

### 2.1 Study population

Study participants were from the China Health and Nutrition Survey (CHNS), which is an ongoing international collaborative longitudinal study aimed at assessing the impact of socio-economic transformation on the health and nutrition status of Chinese residents. The survey began in 1989 and has completed 10-rounds data collection until 2015. The study used a multi-stage random cluster sampling method to recruit 7,200 households and over 30,000 participants from 15 Chinese provinces with significant geographical and economic differences. The objectives and design of CHNS have been elaborated in detail by our previous studies (13, 14). This study follows the guiding principles of the Helsinki Declaration. All processes were approved by the Institutional Review Committees of the University of North Carolina at Chapel Hill (No. 07–1963), and the National Institute of Nutrition and Health, Chinese Center for Disease Control and Prevention (No. 201524) (15, 16). All participants signed informed consent forms before the survey. This prospective cohort study was conducted based on the 1997 to 2015 wave of CHNS.

A total of 33,314 participants were initially selected as the base population in 1997–2015 wave, and baseline of each participant was defined as the date of their first participation in the dietary survey during 1997–2015 wave. The exclusion criteria for the participants were as follows: baseline age was under 18 (*n* = 8,992); participants had no records from the 3 day consecutive 24 h dietary records (*n* = 3,622) or all physical examination data (*n* = 98); participants were diagnosed with cardiometabolic diseases (CMDs) or tumors, or took medicines to treat CMDs at baseline (*n* = 849); participants were included only in a single survey (*n* = 3,789); participants had no dietary data collected through the food weighing method (*n* = 56); participants with an implausible cumulative average of total energy intakes (<800 or >4,200 kcal per day for men; <500 or >3,500 kcal per day for women; *n* = 290); women were breastfeeding or pregnant (*n* = 966). The final analysis of the prospective cohort study included 14,652 participants, including 7,428 males and 7,224 females. The participant process has been shown in Supplementary Figure 1.

### 2.2 Dietary intake assessment

Dietary data were systematically collected using three consecutive 24-h dietary recalls (including two working days and one weekend) at individual level combined with food weighing method at household level. These dietary assessment methods have been validated in previous studies (17–19). Professional interviewers asked each participant one by one about the types, quantities, cooking methods, and dining locations of all food and beverages consumed in the past 24 h. To ensure the accuracy of the data, the research team verified the actual intakes using food weighing method at household level within the same 3-day period (20). The amounts of isoflavones and subtypes consumptions were calculated in reference to the “Chinese Food Nutritional Composition Table” (21, 22). The Planetary Health Diet Index (PHDI) score was calculated based on established method (19). The cumulative average intakes of dietary isoflavones, genistein, daidzein and glycitein, food groups and PHDI score were calculated from baseline to the last day prior to T2DM diagnosis, death, or at the end of follow-up to represent long-term dietary intake patterns. All foods and nutrients were energy-adjusted using the residual method (23). When there was a lack of time for the dietary survey, the questionnaire survey time of the same survey year were used instead.

### 2.3 Ascertainment of T2DM

The main outcome of this analysis was the risk of new onset T2DM in the CHNS cohort between 1997 and 2015 wave, and the diagnosis of T2DM was consistent with previous studies that evaluated the associations between diet and the risk of T2DM using CHNS data (24, 25). Briefly, Professional interviewers collected information about the health status and medical history of participants during each follow-up through questionnaires. And a series of questions were asked to confirm whether participants had ever been diagnosed with diabetes (26): (1) Did the physician give you a diagnosis of diabetes? (2) If yes, how old were you when the physician gave you the diagnosis? (3) Have you ever used the following treatments for glucose control or diabetes, including special diet, weight control, oral medicine, insulin injection, traditional Chinese medicine, folk prescription, qi-gong, and other methods? The participant was identified as diabetes if the answer to any of the three questions was “yes.” Concentrations of fasting blood glucose (≥ 7 mmol/L) and glycosylated hemoglobin (HbA1c, ≥ 6.5%) were measured as supplementary criteria for the confirmation of diabetes in the 2009 wave (27). The cases of diabetes were primarily T2DM due to the exclusion of individuals younger than 18 years old and pregnant women, which excluded type 1 diabetic patients with early onset age and gestational diabetes (28, 29). In case of inconsistencies in the records of different follow-up stages, the records of the first stage would prevail.

### 2.4 Assessment of covariates

The sociodemographic and lifestyle information, including sex, age, province of residence, education level, smoking status, and alcohol consumption, from participants were collected by trained interviewers through validated questionnaires (14). Smokers and drinkers were defined as those who engaged in smoking or drinking behavior during the period from baseline to the end of follow-up. The participants’ height, weight, waist and hip circumferences, and blood pressures were measured according to standard methods via calibrated instruments (30). Body mass index (BMI) was calculated via dividing body weight (in kg) by square of height (in m^2^). Urbanization index in CHNS covered aspects including population density, social services, health, education, economic activity, housing, communication, markets, transportation infrastructure, health infrastructure, and diversity, and the total score ranged from 1 to 120 (14). The northern or southern regions were defined according to the geographical Qinling Huaihe River line (31). Physical activity status was evaluated through intensity and time for activities involving occupation, household, leisure time, transportation, and exercise, which was expressed as metabolic equivalent hours per week (MET-h/week) (32).

### 2.5 Statistical analysis

All statistical analyses were conducted with SAS 9.4 (SAS Institute, Cary, North Carolina, United States) and R software (version 4.5.3, R Statistical Computing Foundation, Vienna, Austria). *p*-value < 0.05 for both tails was considered statistically significant. Baseline sociodemographic, anthropometric and lifestyle characteristics of participants were presented based on tertiles (T) of dietary isoflavones intakes. Continuous variables were presented as mean ± standard deviation (SD) or median (P25, P75), and categorical variables were expressed as N (%). Baseline of each participant was defined as the date of their first participation in the dietary survey during 1997–2015 wave. Differences in baseline characteristics and daily intakes of food groups and nutrients across different tertiles of isoflavones intakes were compared with Kruskal-Wallis rank-sum analysis for continuous variables and Chi-Square test for categorical variables. The effect sizes for baseline characteristics were calculated as the maximum absolute standardized mean differences across tertiles for continuous variables, the maximum absolute standardized differences in proportions across tertiles for binary variables, and Cramér’s V for multicategory variable. The number of person years of follow-up for each participant was calculated from baseline until the last wave of data prior to first diagnosis with T2DM, death, leaving the survey, or until the end of the last survey in 2015, whichever comes first in chronological order (24). The cumulative average values of dietary intake, BMI, urbanization index and physical activity were calculated and analyzed to capture long-term dietary intake patterns and reduce individual differences. These data were not updated following T2DM diagnosis to minimize potential confounding factors caused by changes in dietary intakes or lifestyle behaviors after chronic disease diagnosis.

In this study, Cox proportional hazards regression models with year of follow-up as the time scale was used to assess the associations between dietary intakes of isoflavones, genistein, daidzein, and glycitein with the risk of new-onset T2DM (33). The independent variables were tertiles of dietary isoflavones, genistein, daidzein and glycitein intakes, and the lowest tertile was used as the reference to calculate the hazard ratio (HR) and 95% confidence interval (95% CI) for risk of new-onset T2DM. Proportional hazard assumption was performed prior to Cox regression analysis. Time-dependent variables (time × variable interaction) were incorporated into the Cox regression model for independent variables and covariates that did not meet the proportional hazard assumption. We fitted each independent variable in separate Cox models. Model 1 adjusted for sex (female or male) and age (<50 years, 50–54 years, 55–59 years, 60–64 years, or ≥ 65 years) as confounding factors. Model 2 was a fully-adjusted model, and additionally adjusted for sociodemographic, lifestyle, and dietary confounding factors, including BMI [underweight (<18.5 kg/m^2^), normal weight (18.5–23.9 kg/m^2^), overweight (24–27.9 kg/m^2^), or obesity (≥ 28 kg/m^2^)], region [northern (Beijing, Heilongjiang, Liaoning, Shandong, and Henan) or southern (Shanghai, Jiangsu, Hubei, Guangxi, Hunan, Guizhou, and Chongqing)], urbanization index [low (23.0–49.6), moderate (49.6–75.0), or high (75.0–103.0)], education level [primary (primary school and lower), middle (middle school) and high (high school and above)], alcohol consumption (currently drinking or not), smoking status (currently smoking or not), physical activity status (tertiles), baseline hypertension [normal blood pressure (SBP < 140 mmHg and DBP < 90 mmHg) or hypertension (SBP ≥ 140 mmHg or DBP ≥ 90 mmHg)], total energy intakes (continuous), and PHDI score (continuous). When testing for linear trend, the number of each tertile was included as a continuous variable in the Cox regression model.

Stratified analysis and test for potential effect modifications in the associations between dietary intakes of isoflavones, genistein, daidzein and glycitein with the risk of new-onset T2DM were performed on the basis of sex (female or male), age (<60 years old or ≥60 years old), BMI (<24 or ≥24 kg/m^2^), region (northern or southern), or baseline hypertension history (hypertension or normal blood pressure). Potential confounding factors were the same as in model 2 of the Cox regression model, but the stratification variable was excluded as a confounding factor in the corresponding models. A sensitivity analysis was conducted by excluding cases of cardiometabolic diseases or death that occurred within the initial 2 years of follow-up to estimate the potential impact of reverse causation.

A restricted cubic spline (RCS) based on Cox proportional hazards regression model was conducted using R package “rms” (version 8.1–1) (34) and “survival” (version 3.8–6) (35) with 3 knots (10th, 50th, and 90th), and was performed to test the potential non-linear and dose–response relationships between intakes of isoflavones, genistein, daidzein, and glycitein with the risk of new-onset T2DM. Confounders were consistent with model 2 of the Cox regression models. The median values of the isoflavones, genistein, daidzein, and glycitein were used as a reference in the corresponding RCS analysis.

## 3 Results

### 3.1 Baseline sociodemographic, anthropometric, lifestyle characteristics and food intakes of study participants

This cohort study analyzed data from 14,652 participants. Following a mean follow-up period of 10 years (143,849 person-years) from 1997 to 2015, 1,051 new cases of T2DM were identified. The average age of participants was 45 ± 15 years, with 49.3% females and an average BMI of 22.1 kg/m^2^. Participants with higher isoflavones intakes were more likely to have higher level of education, reside in southern China and areas with medium or high urbanization indices, and no recent smoking or drinking habits and have low or medium level of physical activities, and less likely to smoke and drink alcohol currently (all *p* < 0.05) compared to those with lower intakes (Table 1). Additionally, participants with higher isoflavones intakes were more likely to have higher percent energy of protein and fat and higher intakes of dietary fiber, potassium, vegetables, red meat, eggs, fish, legumes, and saturated oils in comparison with those with lower intakes (all *p* < 0.05; Table 2).

**Table 1:** Baseline sociodemographic, anthropometric, and lifestyle characteristics of 14,652 Chinese adults who participated in the China Health and Nutrition Survey 1997–2015 wave based on tertiles of isoflavones intakes*^a^*.

Variables	All	Tertiles of isoflavones intakes			*p ^b^*	Effect size*^c^*
		T1	T2	T3		
*N*	14,652	4,883	4,884	4,885		
Isoflavones, mg	14.6 ± 23.2	1.1 ± 3.4	11.3 ± 13.1	31.5 ± 30.9	<0.0001	1.38
Daidzein, mg	6.4 ± 10.0	1.3 ± 3.6	5.5 ± 7.0	12.4 ± 13.3	<0.0001	1.13
Glycitein, mg	1.0 ± 1.8	0.3 ± 0.7	1.0 ± 1.3	1.9 ± 2.6	<0.0001	0.84
Genistein, mg	8.3 ± 12.9	1.5 ± 3.8	7.0 ± 8.6	16.3 ± 17.4	<0.0001	1.17
Age, years	45 ± 15	45 ± 16	44 ± 14	46 ± 15	<0.0001	0.11
Female, *N* (%)	7,224 (49.3)	2,362 (48.4)	2,336 (47.8)	2,526 (51.7)	0.0002	0.08
BMI，kg/m^2^	22.1 (20.7, 24.7)	22.0 (20.6, 24.6)	22.2 (20.9, 24.7)	22.3 (20.6, 24.9)	0.0053	0.05
SBP, mmHg	118.7 (110.0, 129.3)	119.3 (110.0, 130.0)	118.3 (110.0, 128.0)	118.7 (110.0, 129.3)	0.0011	0.08
DBP, mmHg	78.0 (70.0, 82.3)	77.3 (70.7, 82.3)	77.3 (70.0, 82.0)	78.0 (70.0, 83.3)	0.70	0.03
Education level, *N* (%)					0.0024	0.02
Primary	6,883 (47.0)	2,287 (46.8)	2,308 (47.3)	2,288 (46.8)		
Middle	4,166 (28.4)	1,435 (29.4)	1,422 (29.1)	1,309 (26.8)		
High	3,603 (24.6)	1,161 (23.8)	1,154 (23.6)	1,288 (26.4)		
Urbanization index, *N* (%)					<0.0001	0.08
Low	4,859 (33.2)	1896 (38.8)	1,693 (34.7)	1,270 (26.0)		
Medium	4,899 (33.4)	1,422 (29.1)	1,649 (33.8)	1828 (37.4)		
High	4,894 (33.4)	1,565 (32.0)	1,542 (31.6)	1787 (36.6)		
Region, *N* (%)					<0.0001	0.16
North	6,101 (41.6)	2,165 (44.3)	2,150 (44.0)	1786 (36.6)		
South	8,551 (58.4)	2,718 (55.7)	2,734 (56.0)	3,099 (63.4)		
Currently smoking, *N* (%)	4,648 (31.7)	1,547(31.7)	1,626 (33.3)	1,475 (30.2)	0.0045	0.07
Currently drinking alcohol, *N* (%)	5,401 (36.9)	1769 (36.2)	1883 (38.6)	1749 (35.8)	0.0100	0.06
Physical activity status, METs-h/week	83.06 ± 108.54	84.94 ± 110.13	87.35 ± 111.14	76.88 ± 103.96	<0.0001	0.10

*^a^* Continuous variables were presented as mean ± SD or median (P25, P75), and the categorical variables were presented as N (%). *^b^* Kruskal–Wallis rank-sum analysis was used in continuous variables and Chi-Square test was used in the categorical variables to test significant differences across different tertiles of intakes of isoflavones. *^c^* Effect sizes were calculated as the maximum absolute standardized mean differences across tertiles for continuous variables, the maximum absolute standardized differences in proportions across tertiles for binary variables, and Cramér’s V for multicategory variables. BMI, body mass index; DBP, diastolic blood pressure; T, tertiles; SBP, systolic blood pressure.

**Table 2:** Baseline daily intakes of nutrients and food groups of 14,652 Chinese adults who participated in the China Health and Nutrition Survey 1997–2015 wave and were included in the analysis with risk of T2DM as outcomes, based on tertiles of isoflavones intakes.

Variables	All	Tertiles of isoflavones intakes			*p-Value^b^*	Effect size*^c^*
		T1	T2	T3		
Total energy (kcal)	2219.8 ± 735.8	2142.7 ± 753.4	2314.9 ± 739.1	2201.9 ± 703.6	<0.0001	0.23
Carbohydrate (%E)	56.2 ± 13.4	56.7 ± 14.3	56.8 ± 13.3	55.2 ± 12.3	<0.0001	0.13
Protein (%E)	12.3 ± 3.0	11.9 ± 3.0	12.1 ± 2.8	12.9 ± 3.0	<0.0001	0.34
Fat (%E)	29.7 ± 12.8	29.7 ± 13.8	29.4 ± 12.8	30.1 ± 11.6	0.0004	0.06
Dietary fiber (g)	11.7 ± 8.8	11.3 ± 7.8	12.0 ± 9.4	11.8 ± 9.0	0.0019	0.08
Na (mg)	5645.1 ± 16455.1	5857.1 ± 26021.8	5696.7 ± 9235.5	5381.6 ± 7071.2	0.0005	0.04
K (mg)	1658.7 ± 850.4	1576.9 ± 697.1	1699.9 ± 981.1	1699.4 ± 843.2	<0.0001	0.16
Na: K ratio	3.7 ± 8.6	4.0 ± 13.2	3.6 ± 5.2	3.5 ± 4.8	0.13	0.05
Whole grains (g)	425.3 ± 165.7	425.2 ± 176.9	438.6 ± 164.1	412.0 ± 154.4	<0.0001	0.17
Vegetables (g)	265.3 ± 145.8	262.8 ± 151.3	266.3 ± 146.9	266.9 ± 138.9	0.0062	0.03
Fruits (g)	26.6 ± 71.0	29.4 ± 74.6	24.8 ± 68.6	25.6 ± 69.4	<0.0001	0.06
Dairy foods (g)	14.8 ± 53.7	17.9 ± 59.2	13.1 ± 50.9	13.5 ± 50.6	<0.0001	0.09
Red meat (g)	66.3 ± 68.9	61.6 ± 68.8	68.1 ± 68.3	69.1 ± 69.4	<0.0001	0.11
Poultry (g)	10.3 ± 25.4	11.0 ± 28.4	10.4 ± 24.9	9.4 ± 22.6	0.0370	0.06
Eggs (g)	23.2 ± 31.4	22.3 ± 31.2	23.9 ± 31.4	23.4 ± 31.5	0.0198	0.05
Fish (g)	19.1 ± 34.2	18.6 ± 35.3	18.8 ± 32.0	19.9 ± 35.2	0.0455	0.04
Legumes (g)	49.0 ± 67.5	13.5 ± 31.7	46.1 ± 56.8	87.5 ± 81.6	<0.0001	1.19
Nuts (g)	3.2 ± 12.3	3.4 ± 12.6	3.3 ± 13.3	2.8 ± 10.9	0.05	0.05
Unsaturated oils (g)	31.8 ± 29.1	32.6 ± 29.4	33.8 ± 31.6	29.2 ± 25.8	<0.0001	0.16
Saturated oils (g)	6.8 ± 18.8	5.6 ± 19.1	7.0 ± 19.3	7.9 ± 17.9	<0.0001	0.12
Added sugars (g)	1.0 ± 5.1	1.2 ± 5.2	0.8 ± 5.3	1.0 ± 4.9	<0.0001	0.07

^a^Continuous variables were presented as mean ± SD. ^b^Kruskal–Wallis rank-sum analysis was used to test significant differences across different tertiles of intakes of isoflavones intakes. ^c^Effect sizes were calculated as the maximum absolute standardized mean differences across tertiles. E, energy; K, potassium; Na, sodium; T, tertiles; SD, standard deviation; T2DM, type 2 diabetes mellitus.

### 3.2 Associations between intakes of isoflavones and subtypes with risk of new-onset T2DM

In models adjusted for age and sex, intakes of isoflavones (model 1; T3 vs. T1: HR = 0.79; 95% CI: 0.68, 0.92; *p*-trend = 0.0051), daidzein (model 1; T3 vs. T1: HR = 0.59; 95% CI: 0.41, 0.84; *p*-trend = 0.0035), glycitein (model 1; T3 vs. T1: HR = 0.86; 95% CI: 0.74, 1.00; *p*-trend = 0.0110) and genistein (model 1; T3 vs. T1: HR = 0.56; 95% CI: 0.39, 0.81; *p*-trend = 0.0017), were inversely associated with risk of new-onset T2DM (Table 3). In fully-adjusted models, intakes of isoflavones (model 2; T3 vs. T1: HR = 0.80; 95% CI: 0.68, 0.94; *p*-trend = 0.0116), daidzein (model 2; T3 vs. T1: HR = 0.60; 95% CI: 0.41, 0.86; *p*-trend = 0.0056), glycitein (model 2; T3 vs. T1: HR = 0.82; 95% CI: 0.70, 0.96; *p*-trend = 0.0133) and genistein (model 2; T3 vs. T1: HR = 0.58; 95% CI: 0.40, 0.84; *p*-trend = 0.0032) were inversely associated with the risk of new-onset T2DM (Table 3).

**Table 3:** Associations between intakes of isoflavones and subtypes and risk of T2DM in Chinese adults who participated in the China Health and Nutrition Survey 1997–2015 wave (*N* = 14,652)*^a^*.

Variables	Tertiles of isoflavones and subtypes intakes			*p*-Trend
	T1	T2	T3	
Isoflavones				
Median (range)	0.46 (0, 4.89)	10.03 (4.89, 16.37)	25.91 (16.37, 257.24)	
Cases (rate, %)	327 (6.70)	361 (7.39)	363 (7.43)	
Person year	40,131	54,772	48,946	
Model 1	1.00 (Ref)	0.73 (0.62–0.84)	0.79 (0.68–0.92)	0.0051
Model 2	1.00 (Ref)	0.71 (0.61–0.82)	0.80 (0.68–0.94)	0.0116
Daidzein				
Median (range)	0.47 (0, 1.88)	3.75 (1.88, 6.07)	9.76 (6.07, 143.14)	
Cases (rate, %)	316 (6.47)	344 (7.04)	391 (8.00)	
Person year	41,224	53,078	49,546	
Model 1	1.00 (Ref)	0.64 (0.51–0.79)	0.59 (0.41–0.84)	0.0035
Model 2	1.00 (Ref)	0.63 (0.50–0.78)	0.60 (0.41–0.86)	0.0056
Glycitein				
Median (range)	0.12 (0, 0.35)	0.66 (0.35, 1.08)	1.71 (1.08, 37.39)	
Cases (rate, %)	335 (6.86)	330 (6.76)	386 (7.90)	
Person year	41,933	52,010	49,906	
Model 1	1.00 (Ref)	0.73 (0.62–0.85)	0.86(0.74–1.00)	0.0110
Model 2	1.00 (Ref)	0.70 (0.60–0.82)	0.82 (0.70–0.96)	0.0133
Genistein				
Median (range)	0.64 (0, 2.44)	4.81 (2.44, 7.73)	12.47 (7.73, 193.14)	
Cases (rate, %)	312 (6.39)	352(7.21)	387 (7.92)	
Person year	40,792	53,555	49,502	
Model 1	1.00 (Ref)	0.64 (0.51–0.79)	0.56 (0.39–0.81)	0.0017
Model 2	1.00 (Ref)	0.63 (0.51–0.79)	0.58 (0.40–0.84)	0.0032

^a^Data were presented as HR (95% CI) estimated by Cox proportional hazards regression models. Rate was calculated as incidence density. Model 1 adjusted for sex and age. Model 2 further adjusted for BMI, region, urbanization index, educational level, physical activity, baseline hypertension, smoking status, alcohol consumption, total energy intake and PHDI. T, tertiles; Ref, reference; T2DM, type 2 diabetes mellitus; PHDI, Planetary Health Diet Index.

Sensitivity analysis excluding participants who were diagnosed with cardiometabolic diseases or died during the first 2 years of follow-up demonstrated similar results with the main analysis. In models adjusted for sex and age, intakes of isoflavones (model 1; T3 vs. T1: HR = 0.80; 95% CI: 0.69, 0.93; *p*-trend = 0.0062), daidzein (model 1; T3 vs. T1: HR = 0.58; 95% CI: 0.40, 0.84; *p*-trend = 0.0036), glycitein (model 1; T3 vs. T1: HR = 0.86; 95% CI: 0.74, 1.00; *p*-trend = 0.0115) and genistein (model 1; T3 vs. T1: HR = 0.55; 95% CI: 0.38, 0.81; *p*-trend = 0.0017) were inversely associated with risk of new-onset T2DM (Table 4). In fully-adjusted models, intakes of isoflavones (model 2; T3 vs. T1: HR = 0.80; 95% CI: 0.69, 0.94; *p*-trend = 0.0142), daidzein (model 2; T3 vs. T1: HR = 0.59; 95% CI: 0.40, 0.86; *p*-trend = 0.0060), glycitein (model 2; T3 vs. T1: HR = 0.82; 95% CI: 0.70, 0.96; *p*-trend = 0.0146) and genistein (model 2; T3 vs. T1: HR = 0.57; 95% CI: 0.39, 0.84; *p*-trend = 0.0034) were inversely associated with risk of new-onset T2DM as well (Table 4).

**Table 4:** Associations between intakes of isoflavones and subtypes and risk of T2DM in sensitivity analysis excluding participants who were diagnosed with cardiometabolic diseases or died during the first 2 years of follow-up (*n* = 14,639)*^a^*.

Variables	Tertiles of isoflavones and subtypes intakes			*p*-trend
	T1	T2	T3	
Isoflavones				
Median (range)	0.46 (0, 4.89)	10.03 (4.89, 16.37)	25.89 (16.37, 257.24)	
Cases (rate, %)	321 (6.58)	358 (7.33)	359 (7.36)	
Person year	40,120	54,766	48,938	
Model 1	1.00 (Ref)	0.73 (0.63–0.85)	0.80 (0.69–0.93)	0.0062
Model 2	1.00 (Ref)	0.71 (0.61–0.83)	0.80 (0.69–0.94)	0.0142
Daidzein				
Median (range)	0.47 (0, 1.88)	3.75 (1.88, 6.07)	9.75 (6.07, 143.14)	
Cases (rate, %)	311 (6.38)	340 (6.97)	387 (7.93)	
Person year	41,215	53,071	49,539	
Model 1	1.00 (Ref)	0.63 (0.50–0.79)	0.58 (0.40–0.84)	0.0036
Model 2	1.00 (Ref)	0.62 (0.50–0.78)	0.59 (0.40–0.86)	0.0060
Glycitein				
Median (range)	0.12 (0, 0.35)	0.66 (0.35, 1.08)	1.71 (1.08, 37.39)	
Cases (rate, %)	330 (6.77)	326 (6.68)	382 (7.83)	
Person year	41,924	52,002	49,899	
Model 1	1.00 (Ref)	0.73 (0.62–0.85)	0.86 (0.74–1.00)	0.0115
Model 2	1.00 (Ref)	0.70 (0.60–0.82)	0.82 (0.70–0.96)	0.0146
Genistein				
Median (range)	0.64 (0, 2.44)	4.81 (2.44, 7.73)	12.47 (7.73, 193.14)	
Cases (rate, %)	307 (6.29)	348 (7.13)	383 (7.85)	
Person year	40,782	53,548	49,494	
Model 1	1.00 (Ref)	0.63 (0.50–0.79)	0.55 (0.38–0.81)	0.0017
Model 2	1.00 (Ref)	0.63 (0.50–0.79)	0.57 (0.39–0.84)	0.0034

^a^Data were presented as HR (95% CI) estimated by Cox proportional hazards regression models. Rate was calculated as incidence density. Model 1 adjusted for sex and age. Model 2 further adjusted for BMI, region, urbanization index, educational level, physical activity, baseline hypertension, smoking status, alcohol consumption, total energy intake and PHDI. T, tertiles; Ref, reference; T2DM, type 2 diabetes; PHDI, Planetary Health Diet Index.

### 3.3 Associations between intakes of isoflavones and subtypes with risk of new-onset T2DM on the basis of potential effect modifiers

**Figure 1:**
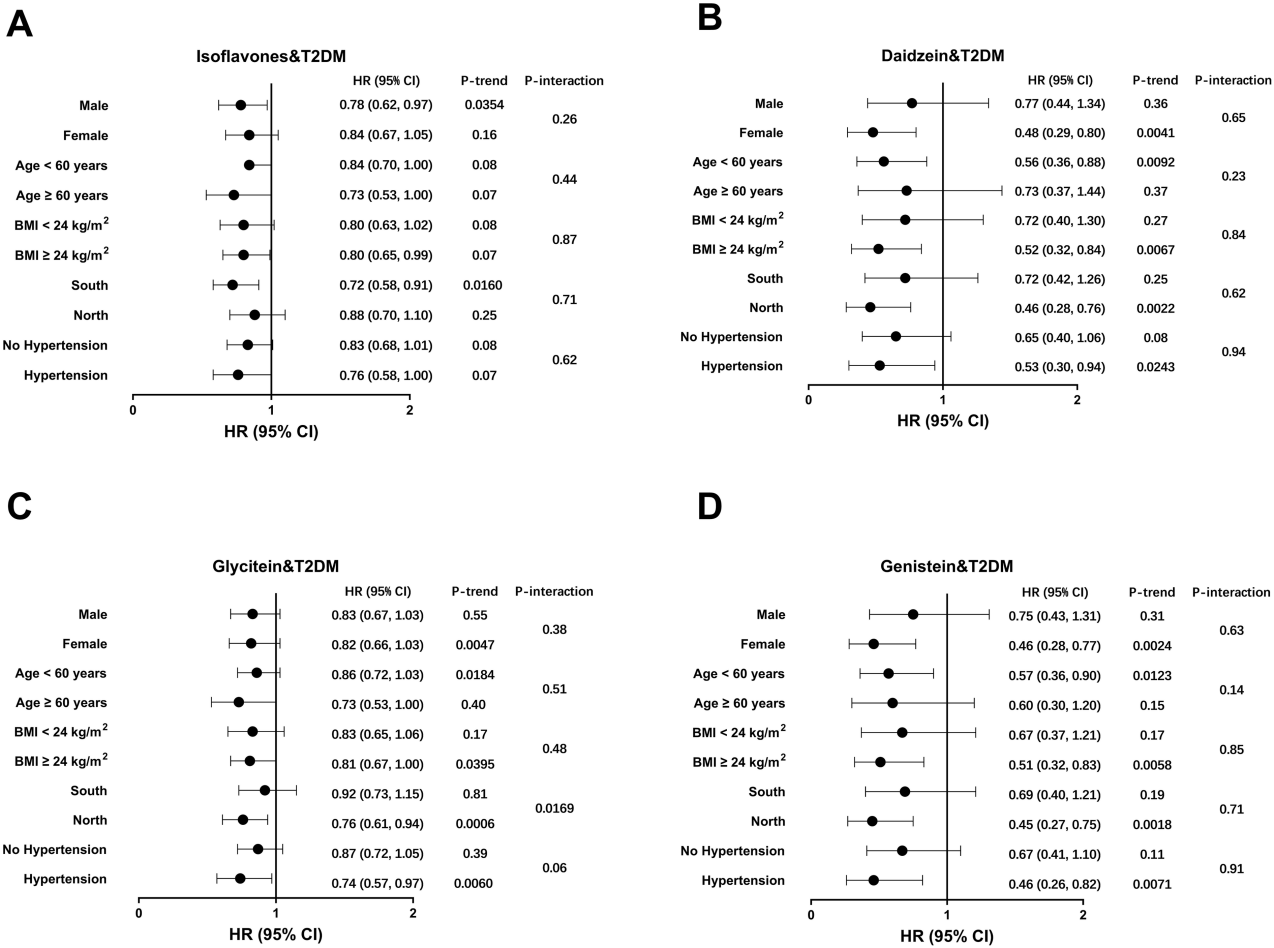
Associations between intakes of isoflavones and subtypes and risk of T2DM in Chinese adults who participated in the China Health and Nutrition Survey 1997–2015 wave (*N* = 14,652), stratified by age, sex, BMI, region and baseline hypertension history **(A)** isoflavones and risk of T2DM, **(B)** daidzein and risk of T2DM, **(C)** glycitein and risk of T2DM, **(D)** genistein and risk of T2DM. Data were presented as HR (95% CI) of T3 vs. T1 estimated by Cox proportional hazards regression models. Models adjusted for sex, age, BMI, region, urbanization index, educational level, physical activity, baseline hypertension, smoking status, alcohol consumption, total energy intake, and PHDI. Stratification variables were not adjusted as confounding factors in the corresponding models. BMI, body mass index; CI, confidence interval; HR, hazard ratio; T, tertiles; T2DM, type 2 diabetes mellitus; PHDI, Planetary Health Diet Index.

There were significant effect modifications of the associations between glycitein intakes and risk of T2DM by region (*p*-interaction = 0.0169) (Figure 1). There was an inverse association between glycitein intakes and risk of T2DM in participants living in northern (T3 vs. T1: HR = 0.76; 95% CI: 0.61, 0.94; *p*-trend = 0.0006) rather than southern China (T3 vs. T1: HR = 0.92; 95% CI: 0.73, 1.15; *p*-trend = 0.81; Figure 1C). There was no evidence of effect modifications of the associations between isoflavones, daidzein, and genistein with risk of T2DM by age, sex, BMI, region, or baseline hypertension (all *p*-interaction >0.05; Figures 1A,B,D).

### 3.4 The nonlinear and dose–response relationships between intakes of isoflavones and subtypes with risk of new-onset T2DM

**Figure 2:**
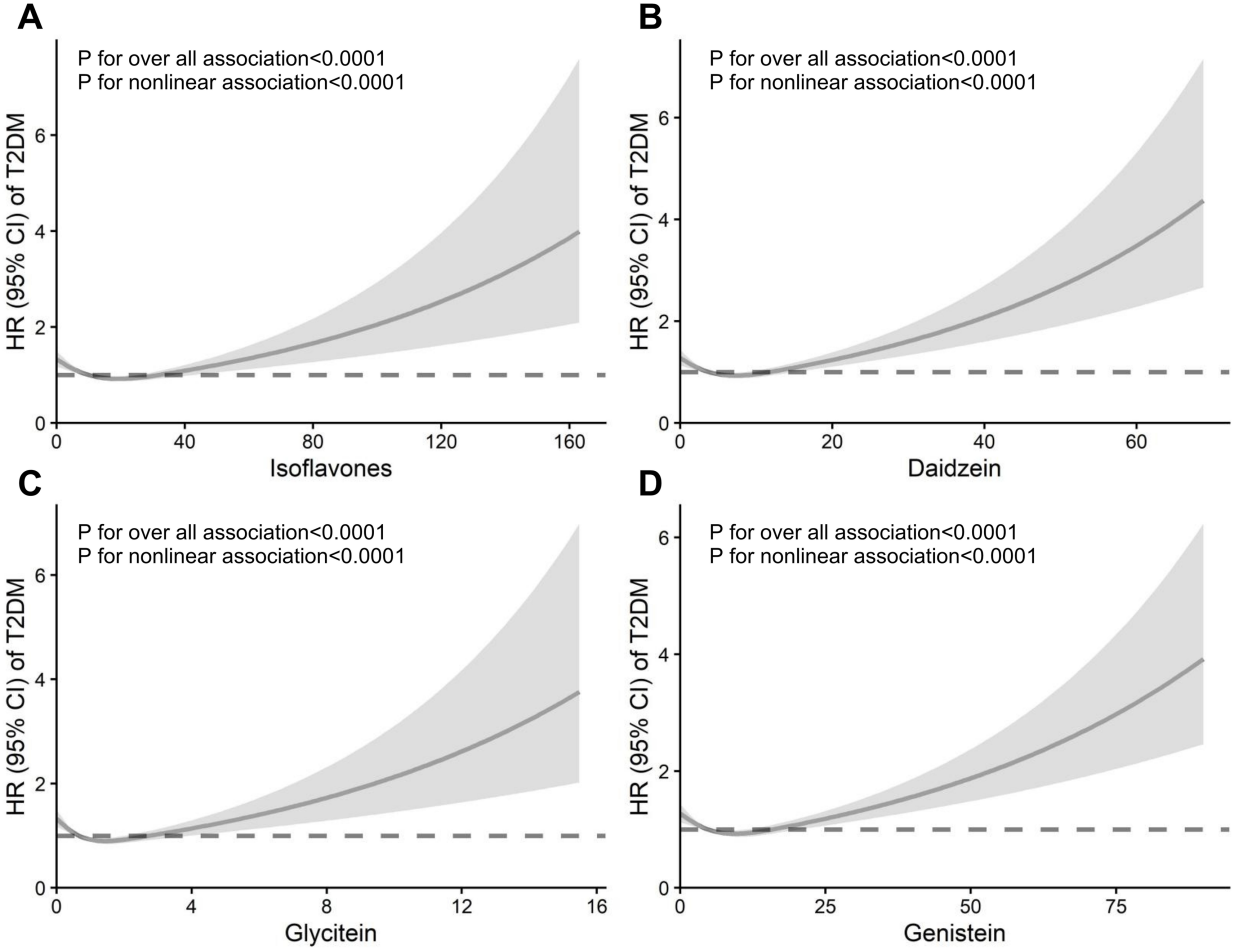
The nonlinear and dose–response relationships between intakes of isoflavones, daidzein, glycitein and genistein with risk of new-onset T2DM (*N* = 14,652) in Chinese adults who participated in the China Health and Nutrition Survey 1997–2015 wave. Gray solid lines and shaded areas were adjusted HRs and 95%CI of T2DM risks associated with isoflavones **(A)**, daidzein **(B)**, glycitein **(C)** or genistein **(D)**. Dotted lines indicated reference lines for no association (HRs of T2DM risk were 1). Models adjusted for age, sex, BMI, region, urbanization index, education level, alcohol consumption, smoking status, physical activity status, baseline hypertension, total energy intakes, and PHDI. Abbreviations: BMI, body mass index; HR, hazard ratio; T2DM, type 2 diabetes mellitus; PHDI, Planetary Health Diet Index.

RCS analysis showed an U-shaped association between isoflavones intake and risk of T2DM (*p* for overall association < 0.0001, *p* for nonlinear association < 0.0001), and isoflavones intake was inversely associated with T2DM risk with the intake range of 10.65–24.58 mg for total isoflavones (HR = 0.92; 95%CI: 0.87, 0.97; Figure 2A), 3.80–8.29 mg for daidein (HR = 0.93; 95%CI: 0.88, 0.98; Figure 2B), 0.70–2.02 mg for glycitein (HR = 0.90; 95%CI: 0.84, 0.96; Figure 2C), and 4.97–11.31 mg for genistein (HR = 0.93; 95%CI: 0.88, 0.98; Figure 2D).

## 4 Discussion

This prospective cohort study with a mean follow-up of 10 years investigated the associations between the dietary intakes of isoflavones and subtypes with risk of new-onset T2DM in a nationwide prospective cohort of Chinese adults. To our best knowledge, this prospective cohort study provides comprehensive evidence that individuals with higher dietary intakes of total isoflavones, daidzein, glycitein, and genistein exhibited a significantly lower risk of developing T2DM. The inverse associations remained robust after adjusting for a wide array of potential confounding factors and in sensitivity analysis. In dose–response analysis, T2DM risk was low with the range of 10.65–24.58 mg isoflavones, 3.80–8.29 mg daidzein, 0.70–2.02 mg glycitein and 4.97–11.31 mg genistein, respectively. These findings alighed with previous epidemiological studies (36–38).

The inverse associations between isoflavones and risk of T2DM may be attributed to the impacts of isoflavones on improving glycemic responses. A RCT study has reported that supplementation with soy protein and isoflavones (132 mg/d) improves fasting insulin and HbA1c concentrations in postmenopausal women with diet-controlled T2DM (39). Another RCT study conducted in male diabetic patients has demonstrated that daily supplementation with 15 g soy protein and 66 mg isoflavones for 3 months significantly improves HbA1c and homeostatic model assessment of insulin resistance (40). A recent network meta-analysis has reported that whole soybeans and isolated isoflavones outperform other soy products in improving glycemic control (41). Isoflavones have estrogen-like structures, and have been reported to by activating key signaling pathways involved in insulin sensitivity and glucose uptake and utilization, such as peroxisome proliferator-activated receptor *γ* (PPARγ) and adenosine monophosphate-activated protein kinase (AMPK) (42, 43). Additionally, isoflavones exhibit potent anti-inflammatory and antioxidant properties, capable of inhibiting the nuclear factor kappa-B (NF-κB) signaling pathway, reducing the production of inflammatory factors such as tumor necrosis factor-*α* (TNF-α) and interleukin-6 (IL-6), and scavenging free radicals (10). These properties of isoflavones collectively result in improvement of pancreatic *β*-cell function, insulin sensitivity and glucose homeostasis, which may contribute to the reduced risk of new-onset T2DM. It is worth mentioning that the ability of individuals to produce equol may be a key factor in regulating isoflavones metabolism *in vivo* (44). Some studies have reported that the inverse association between isoflavones and risk of T2DM only exists in equol-producing individuals, indicating that the process by which gut microbiota converts isoflavones and subtypes into more biologically active forms may be a necessary step in exerting the protective effect (45). Animal studies have also confirmed that S-equol, which is produced from daidzein by gut microbiota, shows protective effect for pancreatic *β*-cell function by activating the cAMP/PKA signaling pathway (46). In addition, *in vitro* studies have explored the interaction between estrogen receptor gene polymorphisms and isoflavones effects, as well as genetic variations in enzymes involved in isoflavones metabolism (e.g., β-glucosidase) (47). Future cohort and interventional studies should consider the role of gene-gut microbiota-isoflavones interactions in affecting the associations between dietary isoflavones with risk of cardiometabolic diseases.

The subtype analysis of isoflavones revealed that dietary daidzein, glycitein, and genistein intakes were inversely associated with risk of new-onset T2DM, consistent with some previous observational studies (36, 48). In accordance with the inverse associations between subtypes of isoflavones with T2DM, a one-year RCT study has demonstrated that daily supplementation with 54 mg of pure genistein improves insulin resistance and fasting blood concentrations of glucose and insulin in postmenopausal women with metabolic syndrome (49). Another 12-week RCT study has also found that 54 mg/d genistein supplementation reduces fasting blood glucose and HbA1c concentrations in postmenopausal women with T2DM (50). However, another RCT study with 50 mg genistein or 50 mg daidzein with 10 g soy protein as interventions in Chinese women with imparied glucose regulation has demonstrated no significantly effects on glycemic profiles (51). The heterogenous findings in RCTs may be attributed to several possible reasons. Participants in China had relatively higher daily isoflavones intake (approximately 10–20 mg/day) from habitual diets in comparison to those from other regions (52), whom may benefit more from additional supplementation with isoflavones or subtypes. The deliverty matrix of subtypes of isoflavones may also contribute to the inconsistent results. Some studies with positive results use aglycone patterns of genistein or other subtypes, which increases absorption rates (42, 49, 50). Inter-individual variations in gut microbiota of participants at baseline contribute to their variability in the ability to convert daidzein into bioactive metabolite equol. However, few studies have conducted stratified analyses based on whether participants are “equol producers.” In addition, the hormonal status of study participants warrants attentions. As phytoestrogens, isoflavones may exhibit more pronounced effects in postmenopausal women with lower estrogen levels, whereas their efficacy might be attenuated in young women or men (53).

Dose–response analysis demonstrated reduced risk of T2DM in participants with total isoflavones intake within the range of 10.65–24.58 mg/d, which were lower than the specific recommended intake levels of isoflavones for Chinese premenopausal and postmenopausal women (55 mg/d and 75 mg/d, respectively) (54). The reason for heterogenous findings may be that Dietary Reference Intakes (DRIs) for isoflavones were primarily derived from studies on cardiovascular disease endpoints, osteoporosis and cancer, rather than T2DM. The dose–response relationships for different health outcomes may vary, as suggested by previous meta-analyses showing that isoflavone intake exhibits nonlinear associations with T2DM risk, with protective effects observed even at moderate intake levels (55). Another dose–response analysis of isoflavones and metabolic disorders in Chinese adults also reported nonlinear trends, further supporting the existence of distinct optimal intake ranges for different health outcomes (56). This nonlinear pattern suggested that the optimal intake range for T2DM prevention may be lower than that for other cardiometabolic endpoints. In addition, the current study included both men and premenopausal women, whereas the DRIs specifically target women or postmenopausal women, who have lower endogenous estrogen levels and may require higher isoflavone doses to achieve comparable metabolic effects due to phytoestrogenic mechanisms. Dose–response RCT studies with rigorous quality controls and full considerations of local dietary habits, metabolic characteristics, gut microbiota and equol-producer are still required to provide scientific evidence for the development of specific recommended intake levels of isoflavones and precision nutrition intervention strategies in Chinese population.

Stratified analysis revealed that the inverse associations between glycitein intakes and T2DM risk were only found in participants lived in northern regions. Isoflavone content shows a certain correlation with latitude and longitude. A study indicates that the total isoflavone content across China generally decreases from north (high latitude) to south (low latitude) (57). Due to glycitein’s relatively low proportion in total isoflavones (about 5–10% in soybeans), variations in the relative content of glycitein versus other isoflavone subtypes may occur across soybean products from different regions. Moreover, there are differences in the processing methods of dishes from northern and southern China, which to some extent may affect the bioavailability of isoflavones and the glycitein content. For example, fermentation significantly increases glycitein levels (58). Given the more frequent consumption of fermented soybean products (such as fermented black soybeans, fermented bean curd, and fermented soybean paste) in northern regions, dietary habits in these areas may influence the protective effects of specific isoflavone subtypes. In terms of pharmacokinetics, compared to other isoflavone subtypes, glycitein is one of the best-absorbed flavonoids, with a 48-h urinary excretion rate (55%) significantly higher than that of daidzein (46%) and genistein (29%) (59), which may account for the observed heterogeneity. While the biological and pharmacokinetic distinctions of glycitein provide plausible explanations for the effect modification patterns, the current evidence for glycitein-specific metabolic benefits remains less extensive than that for genistein or daidzein. Future studies employing purified glycitein monomers in randomized controlled trial settings with adequate statistical power are warranted to validate these subtype-specific findings and to elucidate the underlying mechanisms.

There were several strengths in this study. Previous studies with the same topic utilized case–control or cross-sectional designs. The current study was conducted with long-term prospective cohort data with a mean follow-up of 10 years from adults in 15 provinces and megacities in China, which allowed for investigation of long-term associations between dietary intakes of isoflavones and subtypes with risk of new-onset T2DM. The questionnaires and physical examinations were conducted by trained personnel under strict quality control, ensuring reliable data collection. To confirm accurate recording of dietary intakes, we adopted a dual verification method combining three consecutive dietary recalls at individual level and food weighing method at household level. Additionally, we analyzed potential effect modifications that had received less attention previously, including sex, BMI, age, region, and chronic disease history. The limitations of this study should be noted. Although we adjusted for a wide array of confounding factors, the possibility of other potential residual confounding factors might not be completely ruled out, which was a common issue in observational studies. The dietary recalls might not exclude recall bias. Information on food preparation, processing, and cooking was not included in the analysis. The diagnosis of T2DM relied on self-reported questionnaires rather than clinical testing, which posed challenges in large-scale cohort studies. This study partially overcame this limitation by using fasting blood glucose and HbA1c concentration data from the 2009 cohort for T2DM case confirmation. The information of female reproductive factors, such as menopausal status, age at menopause, history of hormone replacement therapy, or oral contraceptive use was not collected. Given the phytoestrogenic properties of isoflavones and their potential interaction with female hormonal status, future prospective cohort studies with detailed reproductive health information are warranted to better characterize the potential interactions. Additionally, due to the observational nature of this study, potential biological mechanisms were not explored.

## 5 Conclusion

In conclusion, we found inverse associations between dietary intakes of isoflavones and subtypes, including genistein, daidzein and glycitein, with risk of new-onset T2DM in Chinese adults in a natioinwide prospective cohort study with a mean follow-up of 10 years. Region modified the associations between glycitein intake and risk of T2DM. Dose–response analysis demonstrated that the risk of T2DM was low when dietary isoflavones, daidzein, glycitein and genistein intakes were 10.65–24.58 mg, 3.80–8.29 mg, 0.70–2.02 mg and 4.97–11.31 mg, respectively. These findings might add new information to the refinement of dietary reference intake standard of isoflavones, aimed at prevention of T2DM and other cardiometabolic diseases in Chinese population.

## Data Availability

The original contributions presented in the study are included in the article/Supplementary material, further inquiries can be directed to the corresponding authors.
