# Supplementary material for: Associations between dietary isoflavones and subtypes intakes and the risk of new-onset of type 2 diabetes mellitus in Chinese adults: a prospective cohort study from the Chinese health and nutrition survey
Source: Front Nutr. 2026 Jun 16;13:1856176. doi: 10.3389/fnut.2026.1856176 (PMC13314486; doi:10.3389/fnut.2026.1856176)
Supplement: Supplementary file 1 [file Supplementary_File_1.docx]

Supplementary Material

Participants in China Health and Nutrition Survey 1997-2015 wave (N= 33,314)

Participants included in the cohort study (N= 14,652)

Excluded (n =18,662)

- Age< 18 years at baseline (n = 8992)
- Participants had no records from the 3-day consecutive 24-h dietary recalls (n = 3622)
- Participants had no records in all physical examination data (n = 98)
- Participants with CMDs or tumors, or took medicines to treat CMDs at baseline (n = 849)
- Participated in only one wave of the survey (n = 3789)
- Participants had no data from food weighing method (n = 56)
- Implausible cumulative average of total energy intake (n = 290)
- Breastfeeding or pregnant women

(n = 966)

**Supplementary Figure 1.** Selection process for participants in the cohort study in the China Health and Nutrition Survey 1997-2015 wave. Abbreviations: CMDs, cardiometabolic diseases.
