# Supplementary material for: Associations between dietary isoflavones and subtypes intakes and the risk of new-onset of type 2 diabetes mellitus in Chinese adults: a prospective cohort study from the Chinese health and nutrition survey
Source: Front Nutr. 2026 Jun 16;13:1856176. doi: 10.3389/fnut.2026.1856176 (PMC13314486; doi:10.3389/fnut.2026.1856176)
Supplement: Supplementary file 2 [file Table_1.docx]

**Supplementary Table 1.** Sample sizes and incident T2DM cases in the tail ranges of RCS analyses ^a^

| **Exposure** | **Tail range** | **Intake range,mg/d** | **Participants,n** | **Incident T2DM cases,n** |
| --- | --- | --- | --- | --- |
| Isoflavones | ≤P10 | ≤0.12 | 1466 | 90 |
| Isoflavones | ≥P90 | ≥32.99 | 1466 | 109 |
| Daidzein | ≤P10 | ≤0.19 | 1466 | 104 |
| Daidzein | ≥P90 | ≥12.51 | 1466 | 121 |
| Glycitein | ≤P10 | ≤0.04 | 1466 | 107 |
| Glycitein | ≥P90 | ≥2.21 | 1466 | 124 |
| Genistein | ≤P10 | ≤0.32 | 1466 | 111 |
| Genistein | ≥P90 | ≥16.09 | 1466 | 130 |

^a^ Tail ranges were defined as the lower and upper tails at or beyond the boundary knots of the RCS models, corresponding to the ≤10th and ≥90th percentiles of each exposure distribution, respectively.
